# Dental Application of Natural Products

**DOI:** 10.3390/medicines5010021

**Published:** 2018-02-14

**Authors:** Hiroshi Sakagami, Mineko Tomomura

**Affiliations:** 1 Meikai University Research Institute of Odontology (M-RIO), 1-1 Keyakidai, Sakado, Saitama 350-0283, Japan; 2 Division of Biochemistry, Department of Oral Biology & Engineering, Meikai University School of Dentistry, 1-1 Keyakidai, Sakado, Saitama 350-0283, Japan; mineko-t@dent.meikai.ac.jp

**Keywords:** natural products, polyphenols, lignin-carbohydrate complex, glucan, oral cells, QSAR, antitumor, antiviral, antibacterial, receptor

## Abstract

This review article summarizes the recent progress in dental applications of natural products. Catechin gel showed selective antimicrobial activity, whereas the alkaline extract of various plant species rich in lignin carbohydrate complex (LCC) showed much higher antiviral activity than lower molecular weight polyphenols. Mouthwash with the alkaline extract of a plant classified as OTC effectively reduced halitosis. Unexpectedly, many polyphenolic compounds purified from the natural kingdom showed much lower tumor-specificity against human oral squamous cell lines as compared with antitumor agents, although they showed apoptosis-inducing activity. The alkaline extract of bamboo leaf, which exerted various common biological activities with LCC, showed osteogenic activity by stimulating differentiation toward osteoblasts while inhibiting differentiation toward osteoclasts. LCC enhanced the dectin-2 mRNA expression in macrophages, whereas glucan showed anti-osteoblastic action via dectin-1. These data suggest that natural products exert their biological activity by interacting with these molecules.

## 1. Introduction

It is generally accepted that many natural products effectively reduce oxidative stress and show chemoprevention activity in cell cultures and preclinical animal models [1,2,3]. However, due to inefficient systematic delivery and bioavailability, their favorable in vitro and in vivo effects are not reproducible clinically [4]. On the other hand, orally administered products directly contact the oral tissues or cells, and exert their effects without the loss of activity. There is accumulating evidence that the improvement of oral functions by periodontal treatment or the insertion of dentures and implants elevates general health and quality of life [5,6]. This article reviews the recent progress in the study of dental applications of natural products, focusing particularly on polyphenols.

## 2. Classification of Natural Polyphenols

Polyphenols present in the natural kingdom are roughly classified into the following three groups: tannins, flavonoids, and lignin-carbohydrate complexes (LCC) [7]. Tannins are classified into two large groups: hydrolysable tannins (in which a polyalcohol is esterified with a polyphenolic carboxylic acid such as a galloyl, hexahydroxydiphenoyl, valoneoyl, or dehydrohexahydroxydiphenoyl group) and condensed tannins (composed of flavan units, mostly catechin, epicatechin, or their analogs, condensed with each other via carbon–carbon bonds).

Flavonoids are secondary metabolites synthesized from chalcones and categorized into flavonols, flavones, flavanones, isoflavones, pterocarpan, and coumestan. Resveratrol is classified as a stilbenoid.

Lignins are formed through phenolic oxidative coupling processes. Lignin macromolecules are formed by the dehydrogenative polymerization of three monolignols: *p*-coumaryl, *p*-conifery, and sinapyl alchohols. Some polysaccharides in the cell walls of lignified plants are linked to lignin, and recovered as LCC after extraction with an alkaline solution.

## 3. Antibacterial Activity

The oral cavity contains nearly half of the commensal bacterial population of the human body. An increase in the number of these microorganisms may produce systemic diseases such as infective endocarditis and aspiration pneumonia as well as oral infections. In order to suppress the onset of diseases, it is important to control the total numbers of these microorganisms. Green tea catechin showed a bactericidal effect against Gram-negative anaerobic rods, and the slow-release, local delivery of catechin combined with mechanical treatment improved periodontal status [8].

In order to maintain the moistness in the oral cavity of elderly patients who require nursing care, gel-entrapped catechin (GEC) was manufactured by mixing catechins (epigallocatechin, epigallocatechin gallate, epicatechin, epicatechin gallate, gallocatechin, catechin, and gallocatechin gallate) with polysaccharide, dextrin, citric acid, potassium chloride, and stevia [9]. GEC inhibited the growth of the *Actinomyces*, periodontopathic bacteria, and certain tested *Candida* strains, possibly due to the produced hydrogen peroxide, while it did not inhibit the growth of the oral streptococci that are important in the normal oral flora [9].

## 4. Antiviral Activity

Among three representative polyphenols, LCC showed the greatest anti-human-immunodeficiency virus (HIV), influenza virus, and herpes simplex virus (HSV) activity, possibly by directly binding to the viruses [10]. Limited digestion experiments with chlorous acid (which degrades lignin) and sulfuric acid (which degrades carbohydrates) revealed that the lignin moiety, but not the carbohydrate moiety, is essential for anti-HIV activity expression [7]. As expected, sugar-free synthetic lignin, which is achieved using the dehydrogenated polymers of phenylpropenoids (caffeic acid, ferulic acid, *p*-coumaric acid), showed slightly higher anti-HIV activity [7].

The alkaline extract of the leaves of *Sasa senanensis* Rehder (SE) (classified as the third group of over-the-counter drugs), showed antiviral activity comparable to LCC, which was also prepared by alkali extraction and acid precipitation [11]. SE showed synergistic antibacterial activity with isopropyl methylphenol (IPMP) [12], and synergistic antiviral activity with anti-HSV agent (acyclovir) or anti-HIV agent (AZT, ddC) [13]. Furthermore, long-term oral intake of SE progressively reduced the symptoms of lichenoid dysplasia and the salivary concentrations of IL-6 and IL-8 [14]. We have manufactured toothpaste of SE (SETP) containing 26.2 (*w*/*v*%) of SE, IPMP, hydroxyapatite, cellulose gum, humectant, and cleaning, flavoring, stabilizing, and washing agents. Brushing teeth with SETP significantly reduced halitosis in normal volunteers [15].

## 5. Anticancer Activity against Oral Squamous Cell Carcinoma

### 5.1. Polyphenols Show Very Low Tumor-Specificity as Compared with Anticancer Drugs

Most of previous studies of polyphenols have focused on their ability to induce apoptosis in cancer cells, rather than their tumor-specificity [16,17,18]. There is a belief among researchers that apoptosis-inducing activity is a certificate of an anticancer drug. Since there is a morphological similarity between the apoptosis induced by anticancer drugs and that observed during = developmental stages (which eliminates unnecessary or harmful tissues or cells), many people have been engaged in the study of apoptosis. However, we should keep in mind that normal cells are also subjected to apoptosis by anticancer drugs.

In order to quantify the antitumor potency of polyphenols, we established an in vitro assay system for the quantification of tumor-specificity. We used four epithelial human oral squamous cell carcinoma (OSCC) lines (Ca9-22, HSC-2, HSC-3, HSC-4), three mesenchymal human normal oral cells (gingival fibroblast (HGF), periodontal ligament fibroblast (HPLF), pulp cell (HPC)), and two epithelial human normal oral cells (buccal mucosal keratinocytes (HOKs) and primary gingival epithelial cell (HGEP)) [7]. The following two different sets of cells were used: OSCC vs. normal mesenchymal normal oral cells (System 1); OSCC vs. epithelial normal oral cells (System 2). The tumor-selectivity index (TS) was determined by dividing the mean of CC_50_ (concentration that reduced the viable cell number by 50%) against normal cells by the mean CC_50_ against tumor cells. First, we calculated the TS values of anticancer drugs (positive control) using System 1. As expected, many anticancer drugs (docetaxel, 5-fluorouracil, methotrexate, mitomycin C, etoposide, daunorubicin, doxorubicin, SN-38, camptothecin, and gefitinib) showed excellent tumor-specificity (TS = 10–1000), validating this in vitro assay system. On the other hand, polyphenols (LCC, flavonoids, tannins, terpenoids, and their glycosides) (TS = 1~4.8) and antioxidants (sodium ascorbate, gallic acid, catechin, epigallocatechin gallate, chlorogenic acid, daidzein, genistein, quercetin, isoliquiritigenin, kaempferol, resveratrol, and curcumin) (TS = 1.0~4.1) showed disappointingly lower tumor-selectivity, although most of them induced apoptosis in cancer cells [10].

### 5.2. Induction of Keratinocyte Toxicity by Anticancer Drugs

Administration of anticancer agents has been reported to induce skin toxicity [19,20,21,22,23,24,25]. This prompted us to re-evaluate the cytotoxicity and tumor-specificity of anticancer drugs, using System 2. We demonstrated for the first time that anticancer agents such as doxorubicin, daunorubicin, etoposide, mitomycin C, 5-fluorouracil, melphalan, and gefitinib showed comparable cytotoxicity to both epithelial cancer and normal cells, producing very low TS values (TS = 0.1~1.5) [26]. We found that doxorubicin induced apoptosis (loss of cell surface microvilli, chromatin condensation, nuclear fragmentation, and caspase-3 activation) in HOKs [11]. It is therefore urgent to explore new anticancer drugs with less keratinocyte toxicity [26]

### 5.3. Search for New Type Antitumor Agents that Have Higher Tumor-Specificity but Lower Kereatinocyte Toxicity

Sugita’s group synthesized nine groups of compounds to search for new types of anticancer drugs that shower much lower keratinocyte toxicity [26]. Among a total of 133 compounds, (*E*)-3-[2-(4-hydroxyphenyl)ethenyl]-6-methoxy-4*H*-1-benzopyran-4-one (Compound **1**; classified as 3-styrylchromones), (*E*)-3-[2-(4-chlorophenyl)ethenyl]-7-methoxy-2*H*-1-benzopyran (Compound **2**; classified as 3-styryl-2*H*-chromenes) showed the highest tumor-specificity with the least keratinocyte toxicity (TS = 69.0 and 59.9, respectively, in System 1; TS = 204.5 and >85.1, respectively, in System 2) [27]. Compound **1** induced apoptotic cell death in a human OSCC cell line, possibly by downregulating the glycerophospholipid pathway [28]. Quantitative structure−activity relationship (QSAR) analysis demonstrated that the tumor-specificities of Compounds **1** and **2** were well correlated with chemical descriptors related to their molecular size and lipophilicity [27]. Chemical modification of these lead compounds by the introduction of appropriate functional groups is a crucial step towards manufacturing new types of anticancer drugs with reduced keratinocyte toxicity.

## 6. Antiosteoporotic Activity

**Figure 1:**
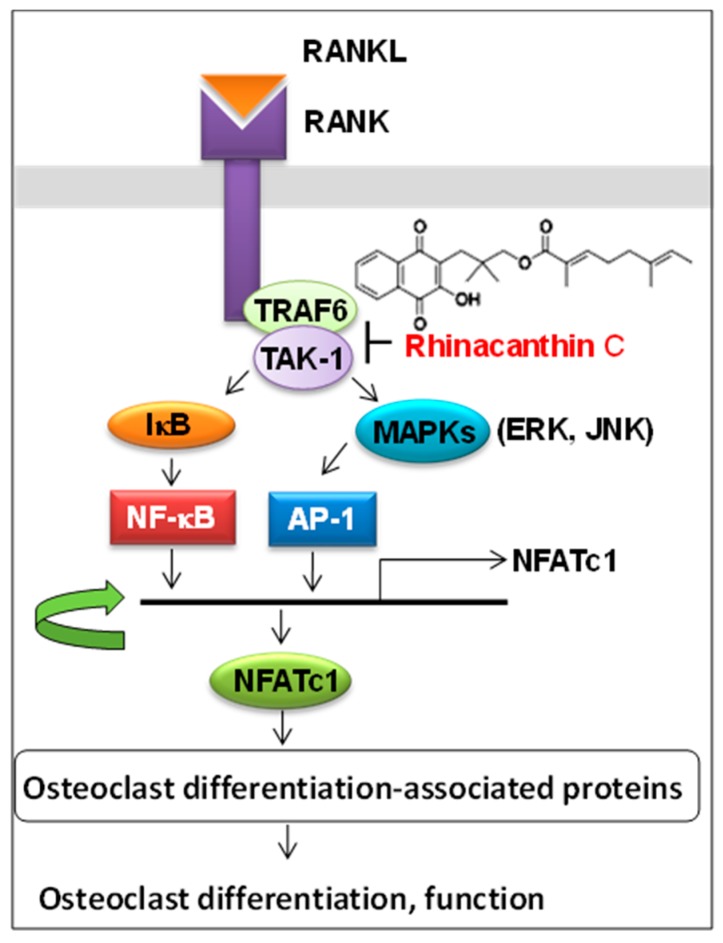
Action point of rhinacanthin C.

Bone homeostasis is maintained by the balance between bone formation by osteoblasts and bone resorption by osteoclasts. When osteoclast differentiation and activation are enhanced, bone structure impairment and bone fracture occur, which are common characteristics of patients with osteoporosis, rheumatoid arthritis, and bone metastatic disease. Osteoporosis-related bone mass reduction accelerates the alveolar bone resorption caused by periodontitis [29]. We found that rhinacanthin C, a naphthoquinone ester isolated from the root and aerial part of *Rhinacanthus nasutus*, potently inhibited the receptor activator of nuclear factor-κB ligand (RANKL)-stimulated osteoclast formation in mouse bone marrow macrophage cultures [30] and in mouse calvarial bone in vivo [31]. Rhinacanthin C inhibited the RANKL-stimulated nuclear factor of activated T cells cytoplasmic 1 (NFATc1) expression, the phosphorylation of ERK, JNK, and NF-κB, and the formation of TRAF6-TAK1 complex [31]. These results suggest that rhinacanthin C inhibits osteoclastogenesis via suppressing RANKL-induced TRAF6-TAK1 association followed by its downstream signaling of the MAPKs/NF-κB/NFATc1 pathway (Figure 1). Rhinacanthin C also suppressed LPS-stimulated osteoclastogenesis and bone resorption [31].

Upon RANKL treatment, mouse-macrophage-like RAW264.7 cells can be differentiated towards TRAP-positive multinuclear osteoclasts [32]. SE completely inhibited the RANKL-induced formation of TRAP-positive osteoclasts and multinuclear cells. The inhibition of mononuclear osteoclast formation was detected at 1% of SE, and complete inhibition of osteoclastogenesis was observed at 2.5% [14].

On the other hand, SE dose-dependently enhanced the alkaline phosphatase (ALP) activity, an early differentiation biomarker of osteogenesis, without significant affecting cell proliferation and cytotoxicity [33]. SE treatment of mouse osteoblastic cell line MC3T3-E1 also stimulated the expression of osteogenic-specific Runt-related transcription factor 2 (Runx2), and other osteogenic biomarkers bone sialoprotein 2 (BSP2) and collagen type 1 protein. Alizarin red and von Kossa staining demonstrated that the terminal step of osteoblast differentiation (calcification) was augmented by SE treatment. This indicated that SE promoted osteoblast differentiation and mineralization.

Taken together, these data suggest that SE reciprocally regulates the cell differentiation of bone-resorbing osteoclasts and bone-forming osteoblasts in vitro. SE may have therapeutic potential for the treatment of bone diseases such as osteoporosis.

## 7. Target Molecules

We previously reported that one of the seven LCC fractions isolated from LEM (Fr4) enhanced the expression of dectin-2 (4.2-fold) and toll-like receptor (TLR)-2 (2.5-fold) prominently, but only slightly modified the expression of dectin-1 (0.8-fold), complement receptor 3 (0.9-fold), TLR1, TLR3, TLR4, TLR9, and TRK13 (0.8~1.7-fold), spleen tyrosine kinase (Syk)b, zeta-chain (TCR)-associated Zap70, Jak2 (1.0~1.2-fold), NFкB1, NFкB2, RELA, RELB (1.0~1.6-fold), NFкB1A, NFкB1B, NFкB1E, NFкBIl2, and NFкBIZ (0.8~2.3-fold) [34]. Fr4 contains lignin precursors such as vanillic acid (25.9 μg/g), syringic acid (25.7 μg/g), *p*-coumaric acid (157.7 μg/g), and ferulic acid (13.4 μg/g) as well as a negligible amount (0.0395 μg/g) of LPS, possibly produced during extraction with NaOH [35]. However, LPS did not affect the expression of dectin-2 or TLR-2. Dectin-2 is a specific receptor for α-mannan, and plays a significant role in the regulation of the protection of the body from *Candida* infection via induction of the differentiation of IL-17-producing T-cells [36]. This suggests that the activation of the dectin-2 signaling pathway may play a significant role in the action of LCC on macrophages [34].

Dectin-1 was identified as a receptor for β-glucan [37]. The dectin-1 agonist curdlan inhibited osteoclastogenesis via the inhibition of NFATc1 through Syk kinase [38,39]. These data suggest that natural products exert their biological activity by interacting with these molecules.

## 8. Future Direction

**Figure 2:**
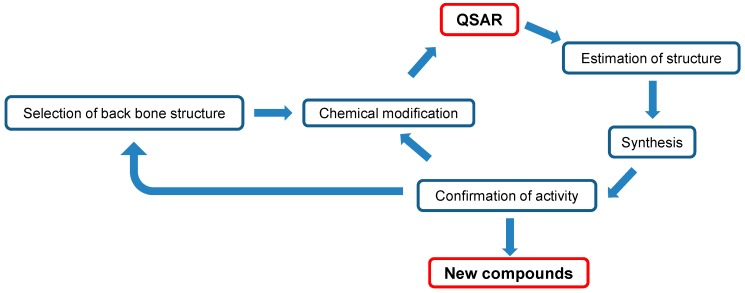
Diagram of manufacturing new antitumor agents.

This review demonstrated that most flavonoids have very low tumor-specificity, although they can induce apoptosis in cancer cells. Even normal keratinocytes are subjected to apoptosis upon doxorubicin treatment, suggesting that apoptosis-inducing activity itself does not guarantee antitumor potential. It is essential to determine the compounds that are highly tumor-specific, but have low keratinocyte toxicity. 4*H*-1-benzopyran-4-ones (chromones) are an important class of oxygenated heterocyclic compounds since the chromone core structure is found in flavones, isoflavones, and 2-styrylchromones. We found that Compound **1** (classified as 3-styrylchromones) and Compound **2** (classified as 3-styryl-2*H*-chromenes) fit into this category. QSAR analysis can be applied to estimate the most potent chemical structures. Synthesis of the estimated structure, reconfirmation of its activity, and repeated cycling of this process will surely yield more active compounds (Figure 2).

We recently found that SE protected amyloid β-peptide-injured neuronal cells [40] and doxorubicin-injured human keratinocytes [26], possibly by its growth-stimulating activity. Hormesis refers to the adaptive responses of biological systems to moderate environmental or self-imposed challenges through which the system improves its functionality and/or tolerance to more severe challenges [41]. Many drugs and radiations produce the stimulatory (i.e., low dose) and inhibitory (i.e., high dose) components of the hormetic dose response [42,43]. It remains to be investigated whether dectin-2 is involved in not only the growth-stimulating action of SE, but also the expression of biological actions of various groups of polyphenols.

We found that the alkaline extraction of green tea leaf, black tea leaf [44], and licorice root [45] produced higher yields of anti-HIV substances, the potency of which was comparable with that of LCC. Thus, alkaline extraction is useful for the efficient utilization of plant resources.

However, there are several problems or limitations concerning the dental applications of natural products. One is the poor absorption of higher molecular weight substances such as LCC and glucans through the stomach and intestine. Actually, we previously reported that only 6% of orally administered [125I] LCC isolated from pine cone extract appeared in the blood of mice after 3 h, and thereafter was excreted into the urine and feces [46]. Since LCC and SE (which have LCC as a major constituent) show excellent antiviral and anti-inflammatory activities, they are recommended to be directly applied to mucous membranes and periodontal tissues for longer periods of time. LCC-vitamin C tablets may be efficacious in improving the condition of HSV-1 patients [47]. On the other hand, cytotoxic lower molecular weight substances can be applicable as a mouthwash or acute treatment. Since many plant materials may be contaminated with LPS from soil bacteria (usually ranging from 10.4 to 18.8 ng/g, sometimes >200 ng/g) [48], extensive washing before extraction and purification is needed.
